# Cytokine Profiles in Patients with Type 2 Diabetes Across Different Durations of the Disease: An Exploratory Cross-Sectional Study

**DOI:** 10.3390/cimb48070651

**Published:** 2026-06-25

**Authors:** Bernard Kordas, Jan Banach, Judyta K. Juranek

**Affiliations:** 1 Department of Human Physiology and Pathophysiology, School of Medicine, Collegium Medicum, University of Warmia and Mazury in Olsztyn, 10-082 Olsztyn, Poland; 2 Faculty of Health Sciences, Jagiellonian University Medical College, 31-008 Kraków, Poland

**Keywords:** type 2 diabetes mellitus, cytokine profile, inflammatory cytokines, chronic low-grade inflammation, immunometabolism, disease duration, interleukins, cross-sectional study

## Abstract

Type 2 diabetes (T2D) is often accompanied by chronic low-grade inflammation, with altered cytokine balance. Although cytokine profiles have often been compared between patients with T2D and healthy individuals, less is known about how they differ among patients with varying disease duration. The aim of this exploratory study was to compare selected pro-inflammatory and anti-inflammatory cytokines in patients with shorter and longer duration of clinically diagnosed T2D. Anonymized surplus serum samples from 18 patients with T2D were analyzed. Patients were divided into two groups according to disease duration: 1–7 years and 8–16 years post-T2D diagnosis. Serum concentrations of six pro-inflammatory cytokines (IL-1β, IL-5, IL-6, IL-8, TNF-α and IFN-γ), three cytokines with anti-inflammatory or immunoregulatory functions (IL-2, IL-4, IL-10), and pro- and anti-inflammatory ratios were measured. All tests were performed using MAGLUMI X8 (Snibe Diagnostics, Shenzhen, China) high-sensitivity chemiluminescent immunoassay according to the manufacturer’s guidelines. Statistical analysis of the data obtained was performed using GraphPad Prism (Boston, MA, USA). The longer-duration T2D group showed higher median concentrations of several pro-inflammatory cytokines, particularly IL-6, IL-8, TNF-α, and IFN-γ, compared with the shorter-duration group. Several values in the longer-duration group exceeded the assay-specific reference intervals provided by the diagnostic platform. Anti-inflammatory and immunoregulatory cytokines showed less consistent differences between groups. Correlation analysis indicated stronger correlations among pro-inflammatory cytokines than among anti-inflammatory or immunoregulatory cytokines. This cross-sectional study suggests that cytokine profiles may differ between patients with shorter and longer durations of T2D, with a pattern consistent with a more pro-inflammatory profile in the longer-duration group. Because of the small sample size, absence of healthy controls, and limited availability of clinical covariates, these findings should be interpreted as descriptive rather than confirmatory and require validation in larger, longitudinal studies with detailed metabolic characterization.

## 1. Introduction

Obesity and type 2 diabetes (T2D) are among the most common health problems worldwide, affecting a large proportion of people around the world. According to the International Diabetes Federation (IDF), one in nine adults worldwide has diabetes [1]. The increasing global prevalence of diabetes has been accompanied by a corresponding rise in the incidence of diabetic complications and related co-morbidities, further worsening the well-being of patients with diabetes as well as burdening the health support system [2]. Studies spanning the last two decades have established that T2D is not only a metabolic disorder but also features an array of multisystemic changes, resulting not only from prolonged hyperglycemia but also from dysregulation of inflammation and cytokine release [3,4,5].

Cytokines belong to the large group of soluble proteins involved in cell–cell signaling and are essential for regulating the immune response [6,7]. Cytokines are divided into different classes or categories depending on their molecular structure, source and other features. Still, the most common and well-established classification is based on their functional properties, dividing them into pro- and anti-inflammatory molecules [7]. Research showed that in T2D, cytokine balance is disturbed, shifting towards increased levels of pro-inflammatory cytokines such as IL-1β, IL-5, IL-6, TNF-α and IFN-γ and towards lower levels of anti-inflammatory cytokines such as IL-2, IL-4 and IL-10 [8,9]. Available evidence indicates that pancreatic β-cell dysfunction and dysregulation of insulin secretion, which exacerbate hyperglycemia, trigger metabolic changes and result in the development of multisystemic diabetic complications [10,11]. Conversely, modulation of excessive inflammatory signaling has been associated with improved metabolic outcomes, slowed disease progression and reduced risk of diabetic complications [3,4,10,12].

Numerous studies have compared cytokine profiles between healthy individuals and patients with T2D. However, less is known about whether cytokine profiles differ among patients with different durations of clinically diagnosed T2D. In the present exploratory cross-sectional study, we analyzed selected pro-inflammatory, anti-inflammatory, and immunoregulatory cytokines in anonymized surplus serum samples from patients with shorter and longer duration of T2D. The aim was to describe differences in cytokine profiles associated with disease duration and provide a basis for future studies using larger, longitudinal cohorts with healthy control groups and detailed metabolic characterization.

## 2. Materials and Methods

### 2.1. Study Design

This was an exploratory cross-sectional study based on anonymized surplus serum samples obtained during routine clinical monitoring of patients with previously diagnosed T2D. The study was designed to compare selected cytokine concentrations between patients with shorter and longer disease duration. The study did not include longitudinal follow-up of the same patients and therefore does not allow direct conclusions regarding within-patient temporal changes. The research team received anonymized material and did not have access to complete patient-level clinical covariates at the time of analysis.

### 2.2. Study Population

Independent healthcare workers anonymized surplus blood serum samples obtained during routine diabetes monitoring, and the anonymized results were sent for our analysis. The samples were obtained from 18 patients with clinically established T2D who were monitored at Specjalistyczne Gabinety, a clinical research and specialist outpatient center in Kraków, Poland, between 2024 and 2025. The study was conducted within the research program approved by the Bioethics Committee of the Faculty of Medicine, Collegium Medicum, University of Warmia and Mazury in Olsztyn, Poland (Resolution No. 31/2023 of 30 November 2023). Anonymized surplus serum samples obtained during routine diabetes monitoring were used, and no additional procedures were performed for this study. T2D diagnosis had been established before sample collection by the patients’ treating physicians as part of routine clinical care. The research team did not independently diagnose T2D and received only anonymized samples and anonymized information available for the present analysis.

Patients were assigned to two groups based on the time elapsed since clinical T2D diagnosis: 1–7 years and 8–16 years. To partially reduce clinical heterogeneity, only samples from patients receiving metformin and without diagnosed diabetic complications or related co-morbidities were included. Detailed patient-level clinical covariates, including HbA1c, BMI, fasting plasma glucose, lipid profile, and blood pressure, were not available in the anonymized dataset at the time of analysis. Available demographic and clinical characteristics of the study cohort are summarized in Table 1.

### 2.3. Cytokine Measurement

Serum concentration of six pro-inflammatory cytokines, i.e., IL-1β, IL-5, IL-6, IL-8, TNF-α and IFN-γ; serum concentration of three anti-inflammatory cytokines, i.e., IL-2, IL-4, IL-10; and pro- and anti-inflammatory ratios were measured. The analysis was performed using high-sensitivity chemiluminescent immunoassays (CLIA) on the MAGLUMI X8 automated analyzer (Snibe Diagnostics, Shenzhen, China), following the manufacturer’s guidelines. The MAGLUMI/Snibe diagnostic platform generated assay-specific reference intervals during automated readout. They were interpreted as technical reference intervals for the respective assays, as provided by the manufacturer. These intervals were not derived from an internally recruited healthy control group and were not independently validated in the present cohort.

### 2.4. Statistical Analysis

Statistical analyses were conducted using GraphPad Prism 10 version 10.5.0 for Windows (GraphPad Software, Boston, MA, USA). Given the small sample size, the analysis was treated as descriptive and exploratory. Cytokine concentrations are presented using median values and visualized in relation to assay-specific reference intervals. Correlations between cytokines were assessed using Pearson’s correlation coefficient and interpreted as descriptive co-variation patterns rather than stable estimates of cytokine relationships.

## 3. Results

**Figure 1:**
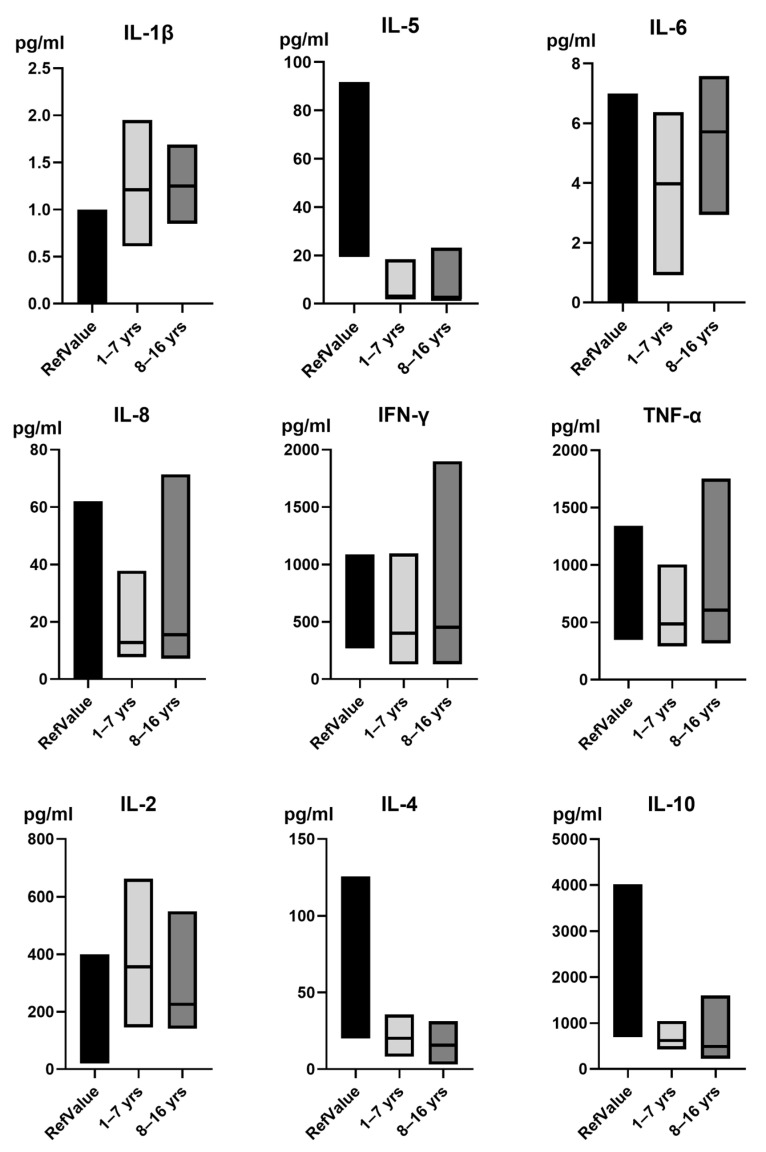
Serum cytokine concentrations in patients with T2D diagnosed 1–7 and 8–16 years (yrs) before sample collection, shown in relation to assay-specific reference intervals (RefValue). Black boxes indicate assay-specific reference intervals. Light gray and dark gray boxes indicate values in the shorter- and longer-duration groups, respectively. Horizontal lines (bars) indicate median values; *n* = 9 per group.

Cytokine concentrations differed between patients with shorter and longer durations of T2D. In the longer-duration group, higher median concentrations or wider upper ranges were observed for several pro-inflammatory cytokines, particularly IL-6, IL-8, TNF-α, and IFN-γ. Values for these cytokines exceeded the assay-specific reference intervals in the longer-duration group, whereas IL-5 remained low in both groups. IL-1β values extended above the assay-specific reference interval, with lower dispersion in the longer-duration group. Among cytokines with anti-inflammatory or immunoregulatory functions, some IL-2 values exceeded the assay-specific reference interval, whereas IL-4 and IL-10 remained close to or below the lower end of the respective intervals (Figure 1, Table 2).

**Figure 2:**
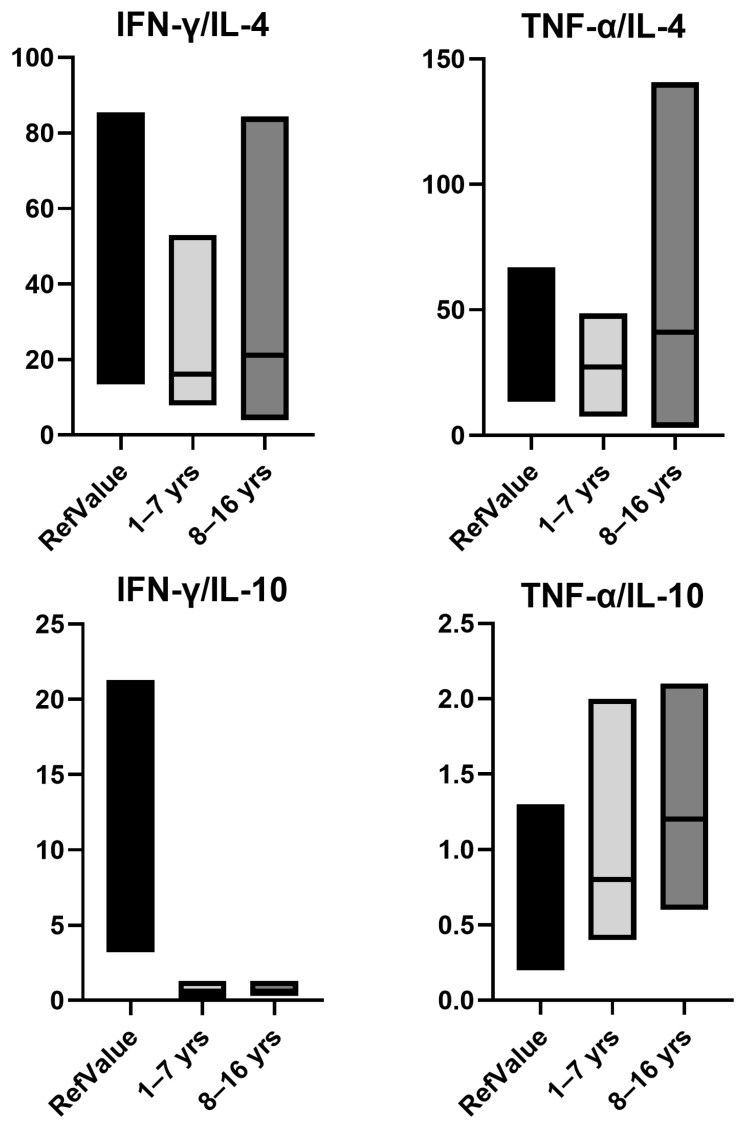
Selected cytokine ratios in patients with T2D diagnosed 1–7 and 8–16 years (yrs) before sample collection, shown in relation to assay-specific reference intervals (RefValue). These ratios were used as descriptive indicators of the balance between selected pro-inflammatory and anti-inflammatory mediators. Black boxes indicate assay-specific reference intervals. Light gray and dark gray boxes indicate values in the shorter- and longer-duration groups, respectively. Horizontal lines (bars) indicate median value; *n* = 9 per group.

Selected cytokine ratios were also compared with assay-specific reference intervals. TNF-α/IL-4 and TNF-α/IL-10 ratios showed values extending above the respective reference intervals, whereas IFN-γ/IL-4 remained within the interval and IFN-γ/IL-10 was below the interval. These findings suggest an imbalance between selected pro-inflammatory and anti-inflammatory mediators. Still, they should not be interpreted as definitive evidence of a progressive immune shift, given the cross-sectional design and small sample size (Figure 2, Table 3).

**Figure 3:**
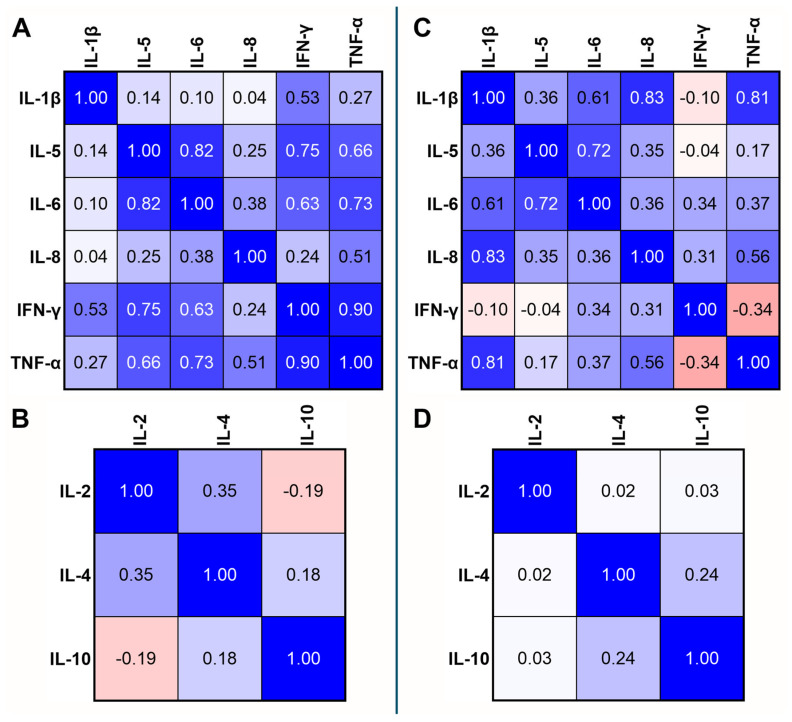
Exploratory correlation matrices. Values represent Pearson correlation coefficients measuring linear correlation between (**A**) pro-inflammatory cytokines at 1–7 years after T2D diagnosis, (**B**) anti-inflammatory or immunoregulatory cytokines at 1–7 years and 8–16 years after T2D diagnosis, (**C**) pro-inflammatory cytokines at 8–16 years after T2D diagnosis, and (**D**) anti-inflammatory or immunoregulatory cytokines at 8–16 years after T2D diagnosis. Color-coded from +1.0 to −1.0.

Exploratory correlation analysis revealed positive correlation patterns among selected cytokine levels in both disease duration groups. In the shorter-duration group, the strongest positive correlations were observed among TNF-α, IFN-γ, IL-5 and IL-6. In the longer-duration group, positive correlations were observed mainly between IL-5 and IL-6, and between IL-1β, IL-8, and TNF-α. No comparable correlation pattern was observed among IL-2, IL-4, and IL-10. Because of the small sample size, these correlations should be interpreted as descriptive co-variation patterns, and not as stable estimates of cytokine relationships (Figure 3).

## 4. Discussion

The present exploratory cross-sectional study compared selected cytokine profiles between patients with shorter and longer durations of clinically diagnosed T2D. The main observation was that the longer-duration group showed higher median concentrations of several pro-inflammatory cytokines, particularly IL-6, IL-8, TNF-α, and IFN-γ. In contrast, anti-inflammatory and immunoregulatory cytokines showed less consistent differences. These findings are consistent with the concept that T2D is associated with chronic low-grade inflammation and altered cytokine signaling [12,17,20,28]. However, the present study does not allow for causal inference or direct assessment of within-patient temporal changes. The results should therefore be interpreted only as descriptive.

The observed between-group differences were not uniform across all cytokines, suggesting that selected inflammatory mediators may vary more clearly than others in relation to T2D duration. However, because the study was cross-sectional and lacked adjustment for metabolic and cardiovascular covariates, these differences cannot be attributed solely to disease duration. An increasing trend was noted for four of the pro-inflammatory molecules, mainly IL-6, IL-8, IFN-γ and TNF-α, extending above the assay-specific reference intervals in the longer-duration group, suggesting a possible increase in systemic inflammation, which has been associated in previous studies with long-term T2D complications as demonstrated by available evidence [4]. Studies showed that both IL-6 and TNF-α are associated with insulin resistance. Specifically, higher levels of IL-6 are correlated with higher levels of glycated HbA [18], which may reflect poorer glycemic control in some clinical contexts, while higher levels of TNF-α correlate with diabetic retinopathy and atherosclerosis [20,21].

IFN-γ and TNF-α may act in partially overlapping or mutually reinforcing inflammatory pathways [22]. Similarly, reports on IL-8 showed that its levels are higher in T2D patients with cardiovascular complications, enhancing immune cell infiltration and activation [19]. IL-1β is one of the crucial cytokines directly involved in beta cell dysfunction already at early stages of both types of diabetes, triggering cell apoptosis and impairing insulin secretion [11,13]. In the present cohort, IL-1β values extended above the assay-specific reference interval in the shorter-duration group, whereas dispersion was lower in the longer-duration group. This observation is consistent with previous studies implicating IL-1β in beta-cell dysfunction and low-grade inflammation in T2D. Conversely, IL-5 levels were below the reference range, with the median value at the lower end in the second group, suggesting that the role of IL-5 in T2D inflammatory changes remains uncertain and requires investigation. Indeed, elevated levels of IL-5 have been considered a risk factor for mild cognitive impairment (MCI) in T2D patients, and MCI symptoms usually occur in later T2D years [14].

IL-2 is a dual-role cytokine that can be pro- or anti-inflammatory, depending on its concentration [23]. In our study, some IL-2 values exceeded the assay-specific reference interval, but this finding should be interpreted cautiously because IL-2 has context-dependent immunoregulatory and pro-inflammatory functions. A study using a rodent model of diabetes showed that low-dose IL-2 improves T2D outcomes by reducing levels of pro-inflammatory cytokines, such as TNF-α and IL-1β, and increasing levels of anti-inflammatory cytokines, such as IL-10 [24], thereby counteracting the consequences of the low-grade inflammation accompanying long-term hyperglycemia. Reports from cell and animal diabetes models showed that IL-4 and IL-10 often act together, suppressing the autoimmune response by Th1 cells, protecting β-cells and lowering overall systemic inflammation [25] and that IL-4, together with IL-10, may protect transplanted islet cells from immune rejection [26]. In the present cohort, IL-4 and IL-10 values remained close to or below the lower end of the assay-specific reference intervals in both disease duration groups. This observation suggests that anti-inflammatory and immunoregulatory cytokines did not show a clear increase accompanying the higher pro-inflammatory cytokine values observed in the longer-duration group. However, this interpretation remains descriptive because no healthy control group was included, and the reference intervals were not independently validated in the present cohort.

Finally, selected cytokine ratios, including IFN-γ/IL-4, TNF-α/IL-4, IFN-γ/IL-10, and TNF-α/IL-10, were used as descriptive indicators of the relative balance between pro- and anti-inflammatory mediators. In this cohort, TNF-α/IL-4 and TNF-α/IL-10 ratios exceeded the assay-specific reference intervals, whereas IFN-γ/IL-4 remained within the interval and IFN-γ/IL-10 was below it.

These observations may be consistent with an altered inflammatory balance in patients with longer T2D duration. However, because no healthy control group was included and the study was not longitudinal, the ratio results cannot determine whether these values differ from those of healthy individuals or whether they reflect progressive changes over time.

## 5. Limitations

This study has several important limitations. The sample size was small, with only nine patients per disease duration group, and no formal sample size calculation was performed. Therefore, the results should be interpreted as descriptive rather than confirmatory. In addition, the study had a cross-sectional design and did not include longitudinal follow-up of the same patients. Consequently, the observed differences between groups cannot be interpreted as direct temporal changes within individual patients. The absence of an age- and sex-matched healthy control group is another important limitation, as the study cannot determine whether cytokine concentrations in patients with T2D differ from those in healthy individuals. Moreover, detailed clinical covariates, including HbA1c, BMI, fasting plasma glucose, lipid profile, blood pressure, and medication adherence, were not available in the anonymized dataset. These variables may influence cytokine concentrations and could represent important unmeasured confounders. Therefore, the observed cytokine differences cannot be attributed solely to T2D duration. Finally, the assay-specific reference intervals used in the study were provided by the diagnostic platform and were not independently established or validated in the present cohort. These intervals should therefore be interpreted as technical reference intervals rather than as a substitute for an internally recruited healthy control group.

Despite these limitations, the study provides descriptive data on cytokine profiles in a clinically defined group of patients with T2D and may guide future research. Larger longitudinal cohorts, including healthy controls and detailed metabolic characterization, are required to validate these observations.

## 6. Conclusions

This exploratory cross-sectional study suggests that selected cytokine profiles may differ between patients with varying durations of clinically diagnosed T2D. The longer-duration group showed a pattern consistent with a relatively more pro-inflammatory cytokine profile, particularly for IL-6, IL-8, TNF-α, and IFN-γ. However, because of the small sample size, lack of healthy controls, absence of longitudinal follow-up, and limited availability of clinical covariates, these findings should be interpreted cautiously. Further validation via larger, longitudinal cohorts with age- and sex-matched healthy controls and detailed metabolic characterization is required to determine whether cytokine profiles change over time during the course of T2D and whether they have clinical or prognostic relevance.

## Figures and Tables

**Table 1:** Demographic and clinical characteristics of patients with type 2 diabetes according to disease duration.

Variable	1–7 Years After Diagnosis	8–16 Years After Diagnosis	Overall
Number of patients	9	9	18
Sex (female:male)	3:6	3:6	6:12
Age (years)	55.8 ± 15.8	58.2 ± 12.4	57.0 ± 13.8
T2D duration (years)	3.9 ± 2.0	11.1 ± 2.9	7.5 ± 4.4
Metformin treatment	yes	yes	yes
Diagnosed complications or comorbidities	none diagnosed	none diagnosed	none diagnosed

Data are presented as mean ± SD unless otherwise indicated. “None diagnosed” indicates that no complications or comorbidities have been diagnosed based on available clinical information. T2D, type 2 diabetes.

**Table 2:** Descriptive summary of cytokine profiles in the studied T2D cohort and context based on selected literature.

Cytokine	Category	Observation in the Cohort	Selected T2D Context
IL-1β [11,13]	Pro-inflammatory	Values extended above the assay-specific RI; dispersion was lower in the longer-duration group	Associated with beta-cell dysfunction and inflammatory signaling
IL-5 [14]	Pro-inflammatory	Low in both groups, mostly below or close to the lower end of the assay-specific RI	Reported in association with inflammatory and neurocognitive changes in T2D
IL-6 [15,16,17,18]	Pro-inflammatory	Higher in the longer-duration group; values extended above the assay-specific RI	Associated with insulin resistance and glycemic control
IL-8 [19]	Pro-inflammatory	Higher upper range in the longer-duration group; values extended above the assay-specific RI	Reported in association with cardiometabolic complications
TNF-α [10,20,21]	Pro-inflammatory	Higher upper range in the longer-duration group; values extended above the assay-specific RI	Associated with vascular complications, including retinopathy and atherosclerosis
IFN-γ [9,22]	Pro-inflammatory	Higher upper range in the longer-duration group; values extended above the assay-specific RI	Involved in inflammatory signaling and may interact with TNF-α pathways
IL-2 [23,24]	Immunoregulatory	Some values exceeded the assay-specific RI	Has context-dependent immunoregulatory and pro-inflammatory functions
IL-4 [25,26]	Anti-inflammatory/ immunoregulatory	Low in both groups, close to or below the lower end of the assay-specific RI	Contributes to anti-inflammatory and immunoregulatory responses
IL-10 [25,27]	Anti-inflammatory/ immunoregulatory	Low in both groups, close to or below the lower end of the assay-specific RI	Contributes to anti-inflammatory and immunoregulatory responses

IL, interleukin; IFN-γ, interferon gamma; RI, reference interval; TNF-α, tumor necrosis factor alpha; T2D, type 2 diabetes. RI refers to the assay-specific reference interval provided by the diagnostic platform. Observations are descriptive and refer only to the studied cohort.

**Table 3:** Descriptive summary of selected cytokine ratios in the studied T2D cohort.

Ratio	Observation	Interpretation
TNF-α/IL-4	Values extended above the assay-specific RI	Pattern consistent with a higher relative TNF-α signal
TNF-α/IL-10	Values extended above the assay-specific RI	Pattern consistent with a higher relative TNF-α signal in relation to IL-10
IFN-γ/IL-4	Generally within the assay-specific RI	No clear deviation from the assay-specific RI
IFN-γ/IL-10	Below the assay-specific RI	Lower IFN-γ/IL-10 ratio; interpretation requires caution

IFN-γ, interferon gamma; IL, interleukin; RI, reference interval; TNF-α, tumor necrosis factor alpha; T2D, type 2 diabetes. RI refers to the assay-specific reference interval provided by the diagnostic platform. Ratios were interpreted descriptively and should not be considered direct evidence of temporal changes in immune function.

## Data Availability

The original contributions presented in this study are included in the article. Additional anonymized data supporting the conclusions of this article may be made available by the authors upon reasonable request, subject to ethical, legal, and privacy-related restrictions.

## Abbreviations

The following abbreviations are used in this manuscript:

BMI	Body mass index
CLIA	Chemiluminescent immunoassay
HbA1c	Glycated hemoglobin A1c
IDF	International Diabetes Federation
IFN-γ	Interferon gamma
IL-1β	Interleukin 1 beta
IL-2	Interleukin 2
IL-4	Interleukin 4
IL-5	Interleukin 5
IL-6	Interleukin 6
IL-8	Interleukin 8
IL-10	Interleukin 10
MCI	Mild cognitive impairment
RI	Reference interval
T2D	Type 2 diabetes
TNF-α	Tumor necrosis factor alpha
